# The Powdered Root of *Eurycoma longifolia* Jack Improves Beta-Cell Number and Pancreatic Islet Performance through PDX1 Induction and Shows Antihyperglycemic Activity in db/db Mice

**DOI:** 10.3390/nu12072111

**Published:** 2020-07-16

**Authors:** Chi-Hao Tsai, Te-Chao Fang, Po-Lin Liao, Jiunn-Wang Liao, Yen-Ju Chan, Yu-Wen Cheng, Ching-Hao Li

**Affiliations:** 1 Department of Physiology, School of Medicine, College of Medicine, Taipei Medical University, Taipei 110, Taiwan; d01447001@ntu.edu.tw (C.-H.T.); d119102001@tmu.edu.tw (Y.-J.C.); 2 Graduate Institute of Medical Sciences, College of Medicine, Taipei Medical University, Taipei 110, Taiwan; 3 School of Pharmacy, College of Pharmacy, Taipei Medical University, Taipei 110, Taiwan; 4 Institute of Food Safety and Health Risk Assessment, School of Pharmaceutical Sciences, National Yang-Ming University, Taipei 112, Taiwan; plliao@ym.edu.tw; 5 Division of Nephrology, Department of Internal Medicine, Taipei Medical University Hospital, Taipei Medical University, Taipei 110, Taiwan; fangtc@tmu.edu.tw; 6 Division of Nephrology, Department of Internal Medicine, School of Medicine, College of Medicine, Taipei Medical University, Taipei 110, Taiwan; 7 TMU Research Center of Urology and Kidney, Taipei Medical University, Taipei 110, Taiwan; 8 Graduate Institute of Veterinary Pathology, College of Veterinary Medicine, National Chung Hsing University, Taichung 402, Taiwan; jwliao@dragon.nchu.edu.tw

**Keywords:** diabetes mellitus, *Eurycoma longifolia*, beta-cell, insulin, PDX1

## Abstract

Non-insulin-dependent diabetes mellitus (NIDDM) is a common metabolic disorder worldwide. In addition to the chief feature of long-standing hyperglycemia, dyslipidemia, hyperinsulinemia, and a number of complications develop in parallel. It is believed that an adequate control of blood glucose levels can cause these complications to go into remission. This study was performed to evaluate the antidiabetic activity of *Eurycoma longifolia* Jack (EL) in vivo. The blood-glucose-lowering activity of EL was studied in db/db mice administered crude powdered EL root (25, 50, and 100 mg/kg) orally for eight weeks. At the end of the study, HbA_1c_, insulin, plasma lipid levels, and histopathology were performed. Powdered EL root showed significant antihyperglycemic activity along with the control of body weight. After eight weeks of treatment, both the blood cholesterol level and the glycogen deposit in hepatocytes were remarkably lower, whereas the secreting insulin level was elevated. An improvement in islet performance was manifested as an increase in beta-cell number and pancreatic and duodenal homeobox 1 (PDX1) expression. Neogenesis or formation of new islets from pancreatic duct epithelial cells seen in the EL-treated group was encouraging. This study confirms the antihyperglycemic activity of EL through PDX1-associated beta-cell expansion resulting in an enhancement of islet performance.

## 1. Introduction

Diabetes mellitus (DM) is a metabolic disorder affecting > 500 million people across the globe [1]. Type I DM (also known as insulin-dependent or IDDM) is primarily caused by the destruction of insulin-secreting beta-cells in the pancreatic islets leading to deficient insulin production in the body. On the other hand, type II DM or non-insulin-dependent DM (NIDDM), which accounts for approximately 90% of all DM cases, is a chronic disorder. It begins with an insensitive response of muscles, the liver, and adipose tissues to insulin. Over time, the exhaustion of beta-cell function causes insulinopenia and leads to the development of impaired glucose tolerance. In both NIDDM and IDDM, patients present with prolonged high blood glucose levels accompanied by symptoms such as polyuria, thirst, hunger, and weight loss. If not properly treated, long-lasting hyperglycemia can lead to serious complications such as foot ulcers, diabetic retinopathy, renal failure, and myocardial infarction [2,3]. Over the past decades, the prevalence of NIDDM has been rising continuously in various populations and age groups including young people. Additionally, the prevalence of NIDDM in low-to-middle income countries is more than that in developed countries [4,5,6]. Epidemiological data indicate that the increase in the prevalence of NIDDM occurs in parallel with obesity, consumption of high-sugar/high-calorie diet, physical inactivity, genetic susceptibility, and smoking, which are the risk factors for NIDDM [7,8,9,10]. It has been estimated that DM will be the seventh-leading cause of death in 2030 and will impose a huge socioeconomic burden [6]. 

*Eurycoma longifolia* Jack (Simaroubaceae family; EL) is an indigenous shrub growing in the sandy soil of Southeast Asia. It is also called “Tongkat Ali”, which is literally attributed to its long twisted root. An aqueous decoction of its roots has been used by the indigenous people for centuries as a folk medicine for several diseases, especially intermittent malarial fever. It is currently consumed as a dietary supplement and sold as different preparations such as drinks, capsules, and tablets (prepared by the addition of the crude powdered roots or extracts) [11,12]. According to the registration database of the National Pharmaceutical Control Bureau of Malaysia, over 384 such products are available commercially in the nutraceutical market. These products were mainly consumed by men as an aphrodisiac for not only enhancing libido, but also improving physical strength [13,14,15]. On the other hand, studies have supported the benefits of EL products in maintaining blood pressure along with exerting other activities, such as anti-osteoporosis, immune regulation, stress relieving, and anticancer activities [11,16,17,18,19,20]. Various phytochemicals have been extracted from the root of *E. longifolia,* including quassinoids, alkaloids, terpenes, polyphenols, high molecular weight polysaccharides, and glycoproteins. Pharmacological studies have shown that eurycomanone, the principal quassinoid, was effective for improving testosterone production, motility [13], and has anti-inflammatory activity [21] and antiproliferative activity in various types of cancer cells [22,23]. In addition, novel polysaccharides purified from *E. longifolia* show unique immunomodulatory activities that involve the enhancement of phagocytosis and cytokine secretion by RAW264.7 cells [17]. Traditional herbs are currently used as alternative/complementary medicine to regulate blood sugar. The present study aimed to determine the efficacy of the “crude powdered EL root” in preventing hyperglycemia in db/db mice. 

## 2. Materials and Methods

### 2.1. Chemicals and E. longifolia (EL) Powdered Root Preparation

The EL root powder used in this study was obtained from *E. longifolia* harvested in Malaysia and processed by Exclusive Mark (M) Sdn Bhd, Malaysia as described earlier [24]. Briefly, the roots (>3 inches in diameter) of 4-year-old plants were cropped. After cleaning, the roots were sliced, dried in an oven at 110 °C oven for 2 h, and then ground to a fine powder. The root powder and the voucher specimen (No. **TMU2012-01**) were preserved in evacuated bags, and stored in the School of Pharmacy, Taipei Medical University. The dry powder was resuspended in sterile water at the time of use. All reagents were obtained from Sigma Aldrich unless otherwise specified. 

### 2.2. Animal Husbandry

The C57BL/6J mice (8-week-old, male) and db/db mice (BKS.Cg-Dock7m^+/+^ Lepr^db^/JNarl, 8- to 12-week-old, male) were acquired from the National Laboratory Animal Center (Taipei, Taiwan). Before beginning the experiments, the mice were routinely acclimated for one week in the animal house in Taipei Medical University. The animals were fed a standard laboratory diet and given water ad libitum. All animal experiments were approved by the Animal Welfare Committee of Taipei Medical University (LAC2016-0168; LAC2020-0060).

### 2.3. Animal Experiments

The diabetic mice were randomized into four groups: control group (sterile water) and EL-treated groups (25, 50, and 100 mg/kg). The experimental doses were based on the results of the pilot study. The dosing volume was 10 mL/kg. Each mouse orally received vehicle or powdered EL root suspension once daily for eight weeks. Mortality rate and abnormal signs were monitored daily. Body weight was recorded weekly, and the blood glucose level was measured at weeks 0, 1, 2, 4, and 8 post dosing. Before the measurement of blood glucose, the mice were made to fast for 16−20 h. At the end of the study, the animals were sacrificed with an overdose of isoflurane. Venous blood samples were collected and centrifuged at 3000 rpm for 15 min to separate the serum, which was then stored at −80 °C for the measurement of insulin levels. The organs (heart, liver, spleen, lung, kidney, brain, and pancreas) were isolated and fixed in 10% neutral-buffered formalin prior to sectioning and embedding. 

### 2.4. Oral Glucose Tolerance Test (OGTT)

An Oral Glucose Tolerance Test (OGTT) was performed at week 7 after the initiation of the study. Briefly, the diabetic mice were made to fast for 16–20 h and provided 1 g/kg of glucose by gavage. Blood glucose levels were analyzed at baseline and 30, 60, 90, 120, and 180 min after glucose administration. Blood samples (5 µL) were obtained from the tail veins and immediately used for glucose estimation by a one-touch electronic glucometer (AccuChek, Roche Diagnostic, Indianapolis, IN, USA).

### 2.5. Serum Biochemistry

The lipid profile (total cholesterol and triglyceride) of the serum samples was determined by commercial chips and a biochemical analyzer (VetTestTM, IDEXX, Westbrook, ME, USA).

### 2.6. Insulin Quantification and Beta-Cell Function Evaluation

Insulin concentration in the serum samples was quantified by using an enzyme-linked immunosorbent assay kit (Mercodia, Sweden). Briefly, the serum samples were incubated with peroxidase-conjugated anti-insulin antibodies and anti-insulin antibodies bound to microplates for 2 h at room temperature with shaking. After thorough washing to remove unbound antibodies, bound conjugates were detected by reaction with 3,3’,5,5’-tetramethylbenzidine at room temperature. Then, the reaction was stopped by adding acid to give a colorimetric endpoint that was read spectrophotometrically at 450 nm. The regression curve obtained from the calibrators was used to calculate the insulin concentration of the samples. The homeostasis model assessment (HOMA) was applied for the quantification of insulin resistance and β-cell function. The HOMA-IR and HOMA-β were calculated from the following formula [25].

(1) HOMA-IR index = insulin (μU/mL) × glucose (mmol/L)/22.5

(2) HOMA-β index = 20 × insulin (μU/mL)/[glucose (mmol/L) − 3.5]

### 2.7. Determination of Glycated Hemoglobin (HbA_1c_)

The percentage of hemoglobin A_1c_ (HbA_1c_) as a percentage of the total hemoglobin was analyzed by using an enzymatic assay kit (Crystal Chem, Elk Grove Village, IL). According to the manufacturer’s instructions, whole blood samples were lyzed in a commercial lysis buffer. Then, 25 µL of the lysate was incubated with 160 µL of a protease reaction mixture at 37 °C for 5 min, allowing the digestion and release of glycated valines from the beta chain of hemoglobin. The glycated valines are specific substrates for fructosyl valine oxidase; these reactions also produce hydrogen peroxide. Finally, the hydrogen peroxide produced was measured by the addition of 70 µL peroxidase and chromagen solution. After 3 min incubation at 37 °C, the optical density was recorded at 700 nm. The HbA_1c_ level in the sample was interpolated using a calibration curve, which was generated by plotting the mean absorbance values of the calibrators.

### 2.8. Histopathological Examination and Immunohistochemical (IHC) Staining

For histopathological examination, formalin-fixed organ specimens were dehydrated using alcohols, cleared in xylene, and then embedded in paraffin. The paraffin sections were 5-μm-thick and stained with hematoxylin and eosin by following the standard protocol. Pathological changes were examined and scored by Prof. Liao Jiunn-Wang (Veterinary Pathologist, Graduate Institute of Veterinary Pathology, National Chung Hsing University, Taichung, Taiwan). Pancreatic islets were imaged and their diameters measured by using Image Eye software (FMJ Software, Stockholm, Sweden).

To observe insulin-secreting beta-cells and pancreatic and duodenal homeobox 1 (PDX1)-expressing cells, pancreatic slices were de-paraffinized and re-hydrated. After antigen retrieval and non-specific antigen masking, the specimens were incubated with primary antibodies (anti-C-peptide, OriGene Technologies Inc., Rockville, MD, catalog AP02083SU-N; anti-PDX1, Bioss Antibodies, Woburn, MA, catalog bs-0923R; anti-proglucagon, Cell Signaling, catalog 8233) diluted in phosphate-buffered saline (PBS) containing 1% BSA overnight at 4 °C, followed by the addition of horseradish peroxidase-coupled secondary antibodies and 3,3’-diaminobenzidine (DAB) to set up a colorimetric reaction. The specimens were then processed routinely by washing, dehydration, counterstaining, and mounting. The peroxidase-labeled specimens were observed under a Nikon light microscope equipped with Polychrome-III camera (YC technology, New Taipei City, Taiwan) and Image Eye software.

### 2.9. Statistical Analyses

Data are expressed as mean ± standard error of the mean (S.E.M.). All statistical analyses were performed using SPSS software (DrMarketing Co. Ltd., New Taipei City, Taiwan). One-way analysis of variance (ANOVA) followed by Dunnett multiple comparisons was used and *p* < 0.05 was considered statistically significant.

## 3. Results

### 3.1. E. longifolia (EL) Reduced Fasting Blood Glucose Levels in Diabetic db/db Mice

The antihyperglycemic activity of powdered EL root was evaluated by using db/db mice that had a gene mutation in the leptin receptor. The db/db mice displayed early insulin resistance and susceptibility to obesity, hyperglycemia, and diabetic dyslipidemia within six weeks of age [26]. In the control mice, the fasting blood glucose level was > 300 mg/dL throughout the experiment, and the mice showed mean body weight gain every week. However, the mice in the powdered EL root-treated groups (25, 50, and 100 mg/kg) showed no significant changes in mean body weight compared with the mice in the control group (Table 1). 

The effects of powdered EL root on fasting blood glucose level are summarized in Table 2. Treatment with 100 mg/kg of powdered EL root significantly reduced the blood glucose level in one week (37.7% reduction) as compared to that in the control group. At week 8, the mean blood glucose level was 134.6 ± 18.7 mg/dL (64% reduction). Similarly, a significant reduction in the blood glucose level was induced with 50 mg/kg of powdered EL root at weeks 2, 4, and 8, as compared to the baseline level (34.4%, 48.5%, and 48.2% reductions, respectively). No statistically significant difference was observed in the blood glucose levels of the 25 mg/kg treated group. Interestingly, no hypoglycemic response was found in the normoglycemic C57BL/6J mice even at 200 mg/kg dosage (Table A1). 

**Figure 1:**
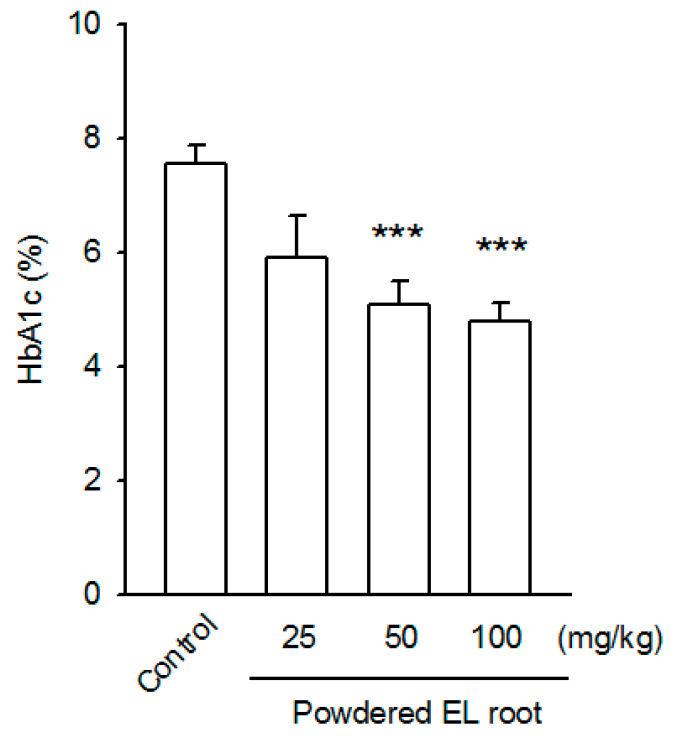
*E. longifolia* reduced glycated hemoglobin (HbA_1c_) content in db/db mice. The administration of 50 and 100 mg/kg EL powdered root for 8 weeks significantly reduced the percentage of HbA_1c_, as compared to the control group. (*** *p* < 0.001, indicates statistically significant difference from the control group).

Glycated hemoglobin (HbA_1c_) is a better indicator of blood glucose level, primarily representing average blood glucose level over the last three months. The HbA_1c_ level in the db/db mice was 7.55 ± 0.33 (% of total hemoglobin). Administration of 50 and 100 mg/kg powdered EL root for eight weeks significantly reduced the HbA_1c_ level to 5.08% ± 0.41% and 4.80% ± 0.32%, respectively (Figure 1). These data strongly support the antihyperglycemic properties of EL in db/db mice (an NIDDM model).

### 3.2. Effects of Powdered EL Root on Oral Glucose-Loaded Diabetic db/db Mice

**Figure 2:**
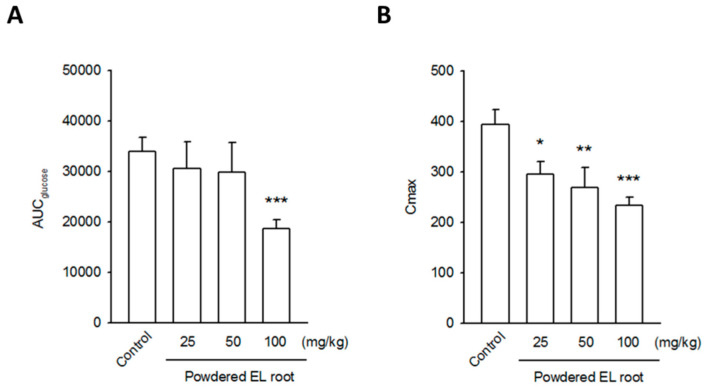
*E. longifolia* changed the kinetic of glucose absorption in db/db mice. The AUC_glucose_ (**A**) and C_max_ (**B**) were calculated as described. A significant reduction in AUC_glucose_ was observed in the high-dose EL-treated group, whereas the notable decrease in C_max_ was found in all EL-treated groups. (* *p* < 0.05, ** *p* < 0.01, *** *p* < 0.001, indicates a statistically significant difference from the control group).

**Figure 3:**
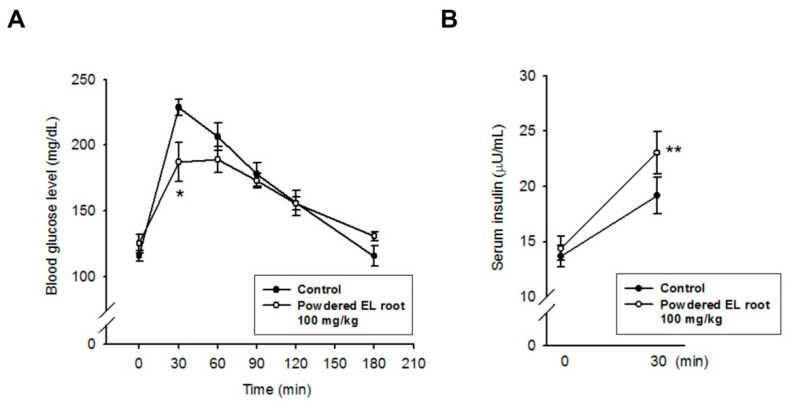
The *E. longifolia*-mediated improvement in glucose tolerance, which coincided with an increase in glucose-stimulated insulin secretion. After administration of powdered EL root for 2 weeks, the C57BL/6J mice were subjected to an OGTT. (**A**) Following the glucose challenge, the EL-treated mice had a lower glycemic peak and a lower area under the curve than the control mice. (**B**) Sera samples were collected at 30 min after the glucose bolus, and the plasma insulin concentration was quantified as described. EL treatment improved glucose-stimulated insulin secretion. (* *p* < 0.05, ** *p* < 0.01, indicates a statistically significant difference from the control group).

Between-group comparisons showed that the baseline glucose level of the control group was significantly higher than that of the 50 and 100 mg/kg treated groups; however, there was no significant difference in the 25 mg/kg treated group. After glucose administration by gavage, a pronounced increase in the blood glucose level was observed within 30 min in all the groups regardless of the treatments. Then, the blood glucose level gradually returned to the baseline in all the groups at 180 min post oral glucose load (Table 3). The area under the curve for glucose (AUC_glucose_) was calculated using the trapezoidal rule. There was no statistical difference in the AUC_glucose_ among the control, 25, and 50 mg/kg treated groups, whereas the AUC_glucose_ significantly decreased in the mice receiving 100 mg/kg of powdered EL root (Figure 2A). The peak of blood glucose concentration (C_max_) was also determined. A notable reduction in C_max_ was found in the EL-treated groups (Figure 2B), which may be attributed to the rapid redistribution of glucose in the tissues. To affirm this finding, the C57BL/6J mice were subjected to an OGTT. Although basal blood glucose and insulin levels did not differ significantly between the control and 100 mg/kg powdered EL root-treated group, during the OGTT, EL-treated mice had a lower glycemic peak 30 min after the glucose loading than the control mice (Figure 3A). Coincidentally, the plasma insulin level was higher in the EL-treated mice than in the control mice (Figure 3B), which suggested that the improved response to the OGTT could be due to an increase in glucose-stimulated insulin secretion.

### 3.3. Powdered EL Root Had Lipid-Lowering Effects in Diabetic db/db Mice

**Figure 4:**
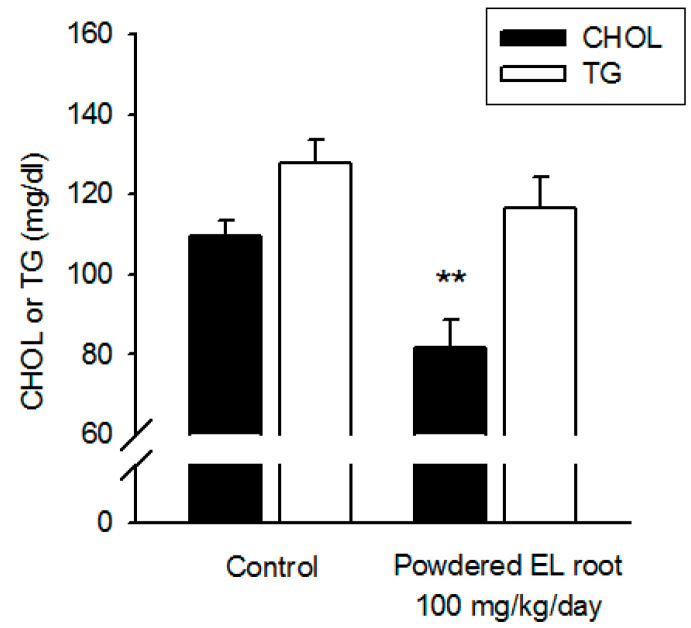
*E. longifolia* manifested a lipid-lowering effect in db/db mice. The amount of cholesterol and triglyceride in sera has been measured as described in Materials and Methods. A significant reduction in cholesterol was observed in the high- and middle-dose EL-treated groups, whereas the triglyceride level remained unaltered. (** *p* < 0.01, indicates a statistically significant difference from the control group).

Total cholesterol and triglyceride levels were reported to be significantly increased in the diabetic db/db mice, indicating dyslipidemia [26]. The serum total cholesterol level was remarkably decreased in the groups treated with 50 and 100 mg/kg of powdered EL root (88.4 ± 4.5 and 81.5 ± 7.1 mg/dl, respectively) (Figure 4), whereas the triglyceride level remained unaltered among the powdered EL root-treated groups. These data support the lipid-lowering effects of EL in db/db mice.

### 3.4. Powdered EL Root Reduced Glycogen Accumulation in Hepatocytes but Improved Pancreatic Islet Volume in db/db Mice

**Figure 5:**
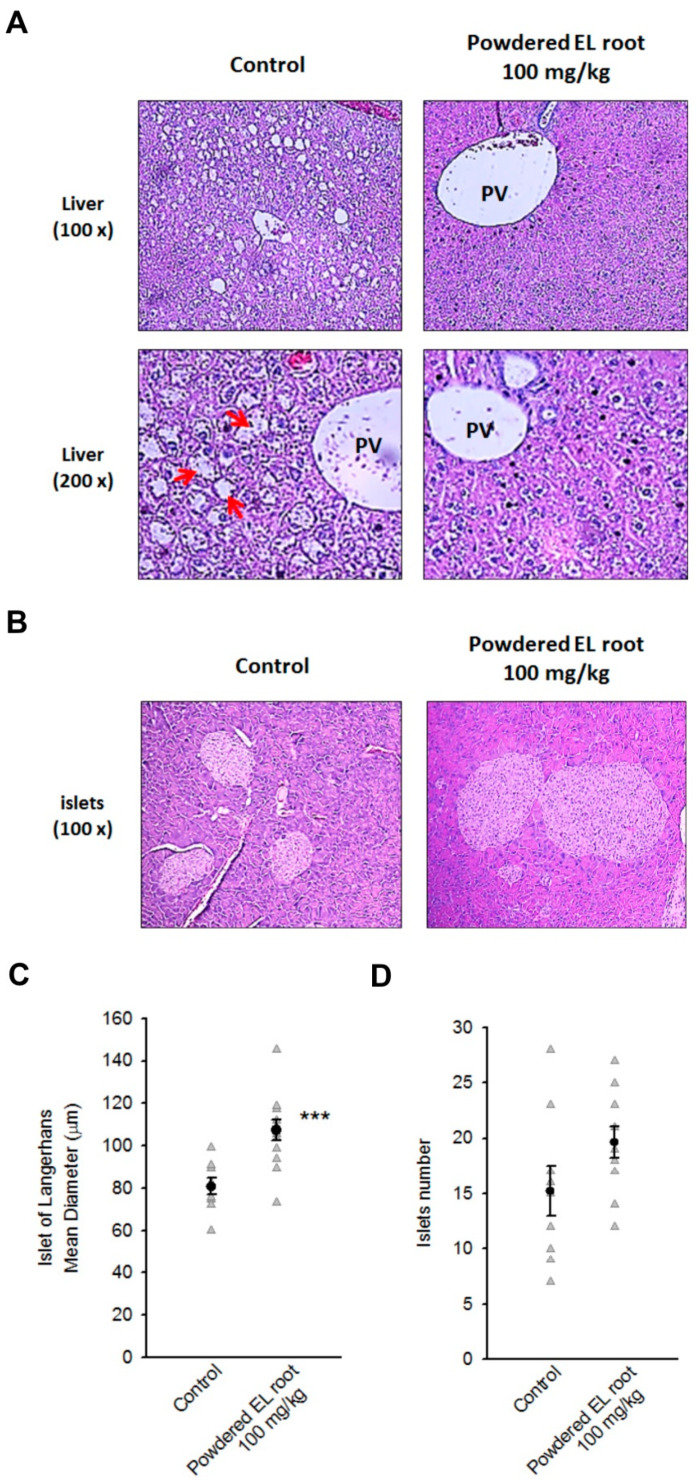
*E. longifolia* reduced glycogen accumulation in the hepatocytes, but improved pancreatic islet volume in db/db mice. (**A**) All db/db mice displayed glycogen hepatopathy. Treatment with EL powdered root obviously alleviated the masses of glycogen particles (arrow) inside the hepatocytes. (**B**, **C**) The representative images and corresponding quantification histogram showed an enlargement of pancreatic islets which was characterized after an 8-week administration of EL powdered root. The data represented the increase in islet volume in the EL-treated mice. (**D**) The number of islets did not differ significantly between the control and EL-treated group (*** *p* < 0.001, indicates a statistically significant difference from the control group).

**Figure A1:**
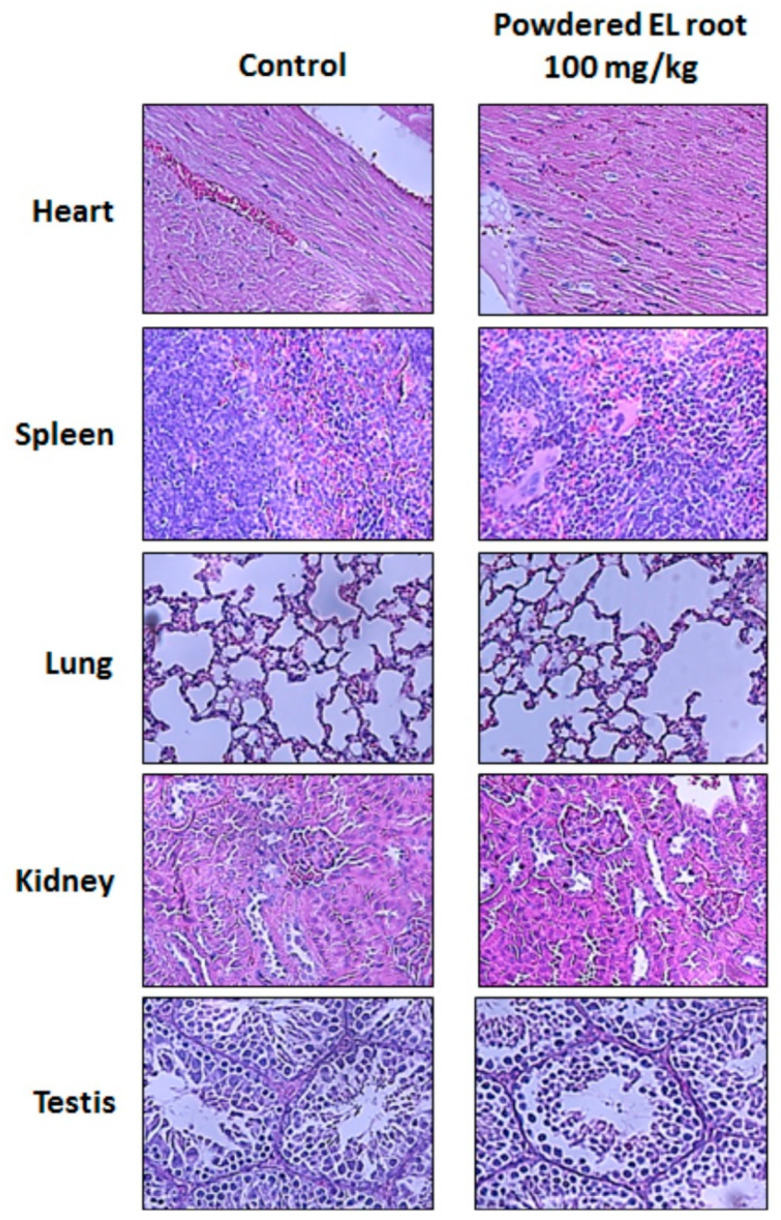
Histopathology examination on EL-treated db/db mice. Representative images showed that after 8 weeks treatment, there were no EL-related pathology signs in heart, spleen, lung, kidney, and testis.

After eight weeks of treatment, there were no EL-related pathological changes in the heart, spleen, lung, kidney, and testis (Figure A1). All db/db mice displayed a diffused, slight to severe glycogen accumulation in their liver, especially the hepatocytes around the portal veins, where the masses of glycogen particles were discrete and compact, a histological feature of glycogen hepatopathy. Treatment of the db/db mice with 100 mg/kg powdered EL root resulted in a significant reduction in glycogen accumulation. The glycogen particles appeared more dispersed within the mass (Figure 5A). On the other hand, the pancreas of the db/db mice showed multifocal, slight to moderate/severe proliferation in the islets (83% incidence) as compared to the normoglycemic C57BL/6J mice. The diameter of the islets of the 100 mg/kg treated group was 107.36 ± 4.80 µm, which was significantly larger than that of the control group (80.75 ± 3.89 µm) (Figure 5B,C). The number of islets was not significantly different between the control group (15.22 ± 2.27) and the 100 mg/kg EL-treated group (19.64 ± 1.40) (Figure 5D). The data signify that treatment with EL might increase the volume (or mass) of the pancreatic islets. Subsequently, islet functions and density of the beta-cells were evaluated.

### 3.5. Powdered EL Root Increased PDX1 Expression and Beta-Cell Number Resulting in an Improvement in Insulin Secretion

**Figure 6:**
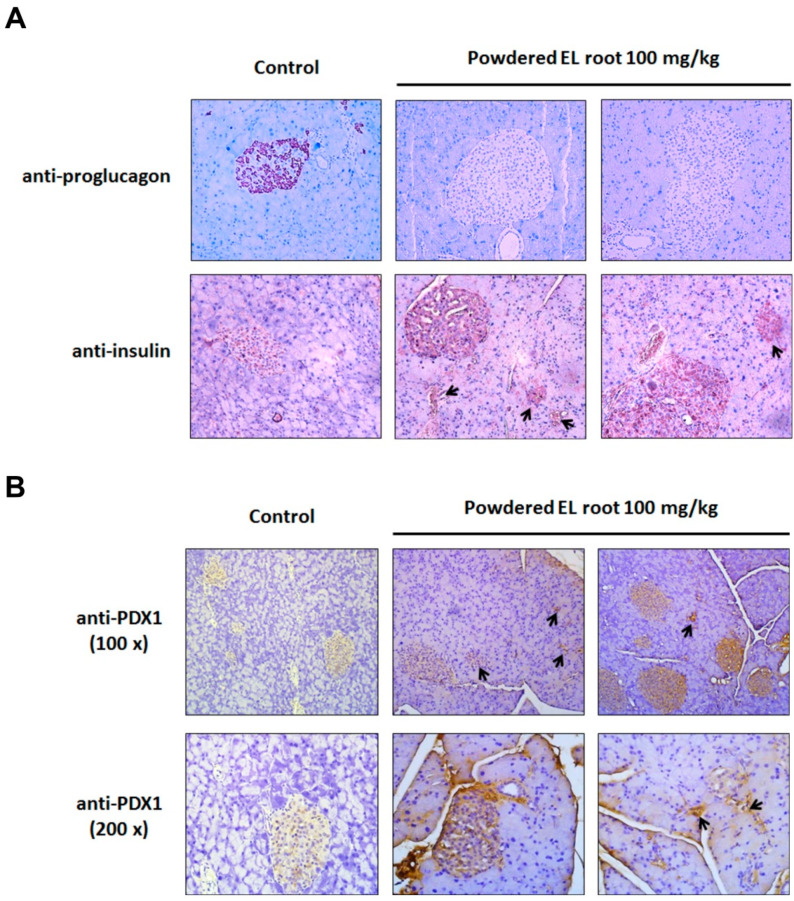
*E. longifolia* increased PDX1 expression and increased β-cell number. Representative images showed the immunostaining of glucagon (**A**, **upper panel**), insulin (**A**, **lower panel**) and PDX1 (**B**) in pancreas slices. (**A**,**B**) The glucagon-producing α-cells were stained brown and nuclei stained blue. A notable reduction in α-cell population was presented in the EL-treated group, as compared to that in the control. The insulin-positive cells are dominantly present inside the islets, but also found in pancreatic ducts. Moreover, a stronger immunoreactivity to insulin was presented in the EL-treated mice, as compared to the control group. (**B**) A similar pattern was found in the immunostaining of PDX1, a biomarker of beta-cell neogenesis. More PDX1-positive cells were stained in islets and pancreatic ducts of the EL treated mice. This finding suggested the regeneration of beta-cells through an induction of PDX1.

The number of alpha-cells and beta-cells was examined by immunohistochemical (IHC) staining. In Figure 6A, a condensed staining of glucagon-expressing alpha-cells was found inside the islets of the control group, whereas a notable reduction in alpha-cell population was found in the EL-treated group. In contrast, insulin-positive cells were predominantly present inside the islets in both the control and EL-treated groups. Furthermore, a strong immunoreactivity against insulin was detected in the pancreatic islets of the EL-treated mice as compared to the control group, suggesting that the increase in islet volume was mainly due to an increase in beta-cell number. It is encouraging that numerous pancreatic ducts were seen along with insulin-positive cells, suggesting neogenesis of beta-cells (Figure 6A, lower panel). We subsequently examined PDX1 expression, a biomarker of beta-cell neogenesis. The data show weak PDX1 staining in the control group. In contrast, the pancreatic islets of the EL-treated groups were notably immunoreactive to PDX1. Furthermore, PDX1 was also expressed within the epithelium of the pancreatic ducts, suggesting new islet formation from the exocrine ducts (Figure 6B). 

**Figure 7:**
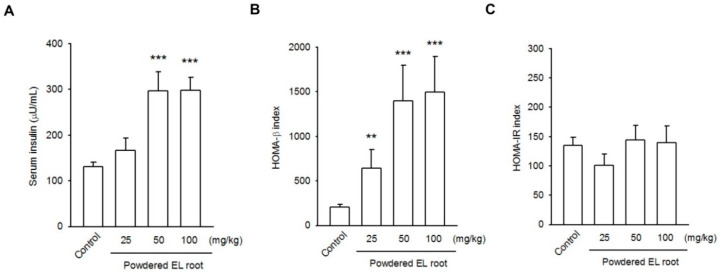
*E. longifolia* enhanced beta-cell performance, resulting in an improvement of insulin secretion. (**A**) The elevations of serum insulin level were detected in the 50 and 100 mg/kg administrated groups. By using a mathematical approach, the HOMA-β index (**B**) and HOMA-IR index (**C**) were used to assessβ-cell function and insulin resistance, respectively. No statistical difference was found in the HOMA-IR index among the control and EL-treated groups. However, HOMA-β was notably increased in the EL-treated groups, as compared to that in the control. These data affirmed that EL may improve islet performance either by an induction of PDX1 or by the increase in beta cell number. (** *p* < 0.01, *** *p* < 0.001, indicates a statistically significant difference from the control group).

Additionally, a significant increase in the serum insulin level was detected in the 50 and 100 mg/kg EL-treated groups (14.00 ± 1.95 and 12.51 ± 1.54, respectively) as compared to that in the control mice (7.75 ± 0.84) (Figure 7A). Next, the insulin resistance and β-cell function were evaluated by a mathematical approach, the homeostasis model assessment (HOMA). No statistically significant difference was found in the HOMA-IR index between the control and EL-treated groups. However, HOMA-β was notably increased in the EL-treated groups compared with the control group (Figure 7B,C). These data prove that EL increased the plasma insulin level by increasing the number of beta-cells.

## 4. Discussion

In IDDM, insulin-producing beta-cells are mistakenly destroyed owing to unknown reasons and this process cannot be prevented by applying existing knowledge. NIDDM is fundamentally caused by insulin resistance and its pathogenesis is strongly associated with obesity and inadequate physical activity. A meta-analysis of the risk of NIDDM and the uptake of different food categories suggested an increased risk with increased consumption of red meat, processed meat, and sugar-sweetened beverages; and a reduced risk with increased consumption of whole grains, fruits, and dairy products [7]. The uptake of excessive calories not only causes fat accumulation in the liver, but also elevates free fatty acid flux from the adipose tissue to the circulation, consequently damaging the beta-cells (lipotoxicity) and exacerbating insulin resistance [27]. Available glucose-lowering drugs are unable to control hyperlipidemia. Furthermore, the peroxisome proliferator-activated receptor-gamma (PPARγ) agonists (e.g., rosiglitazone) are reported to promote adipogenesis [28,29]. For this reason, there is a growing interest to identify novel bioactive compounds that have the capacity to increase insulin sensitivity and lower lipid levels simultaneously. A multitude of functional foods and/or food supplements have been developed for this purpose. EL is a popular traditional herb that has a potent medicinal value. In this study, after an eight-week oral administration of crude powdered EL root, the hyperglycemic db/db mice showed significantly lower fasting blood glucose and HbA_1c_ levels, and the circulating level of cholesterol was also decreased. No hypoglycemic effect was found in normal mice. These results were dose-dependent and fully support the antidiabetic and anti-dyslipidemic potential of EL. As a consequence of long-standing hyperglycemia, the prevalence of liver complications in the diabetic population is estimated to be 17−100% [30], with hepatic steatosis and glycogenic hepatopathy being the dominant pathologies. Glycogenic hepatopathy is diagnosed by the presence of hepatomegaly and reversible glycogen accumulation in hepatocytes. Although their pathophysiology has not been fully explored, it is believed that adequate glycemic control could alleviate the observed histological abnormalities [31]. Our data confirmed the relationship between the EL-mediated glycemic control and the reduction in glycogen accumulation in the liver. 

Most antidiabetic drugs reduce blood sugar mainly by promoting insulin release from the islets of Langerhans or by improving tissue sensitivity to insulin. Other drugs, such as α-glucosidase inhibitors, inhibit the hydrolysis of carbohydrates after minimizing the absorption of monosaccharides in the intestine. Many medicinal plants have been historically used in complementary and/or alternative medicine to treat hyperglycemia. These plants are enriched in bioactive phytochemicals, such as polyphenols, flavonoids, terpenoids, saponins, carotenoids, alkaloids, and glycosides, which possess antihyperglycemic activity [3]. In general, these phytochemicals successfully improve beta-cell performance, resulting in increased insulin secretion or reduced intestinal glucose absorption. The methanolic extract of EL (50−400 µg/mL) has demonstrated the ability to suppress lipid accumulation during adipocyte differentiation. Moreover, 3T3-L1 adipocytes showed an improved sensitivity to insulin, reflecting a substantial enhancement of glucose uptake [32]. In our study, either a reduced AUC_glucose_ uptake following OGTT or an elevated serum insulin level was demonstrated in the db/db mice treated with powdered EL root. The former might reflect the improvement in insulin sensitivity in skeletal muscle and adipose tissue, leading to rapid glucose absorption and utilization by these tissues. The elevated insulin in the circulation represents the improved performance of the pancreatic islets.

In healthy adults, beta-cell mass occupies approximately 2% of the pancreatic weight. However, patients with both types of diabetes have dramatically reduced beta-cell mass [33,34]. The loss of beta-cell mass may be the result of an increased apoptosis frequency that partially corresponds to glucotoxicity and/or lipotoxicity. Inflammatory cytokines (e.g., interleukin-1beta, tumor necrosis factor-α, and interferon γ) and their downstream effectors (e.g., inducible nitric oxide synthase and free radicals) are also the executors of cell death, leading to islet atrophy. As mentioned earlier, phytochemicals obtained from EL are potent nuclear factor-kappa B inhibitors [35] that significantly inhibit the in vitro induction of cyclooxygenase-2, inducible nitric oxide synthase, and interleukin-6 [36,37]. These results suggest that treatment with EL might suppress tissue inflammation and prevent cell death, which may be the underlying reason for improved islet survival and functions. 

In our study, a remarkable increase in the diameter of islets (relative to islet mass) was observed in db/db mice, which had also been reported earlier [38]. Interestingly, islet mass was significantly larger after the administration of powdered EL root, indicating beta-cell proliferation. This change was also responsive to elevated insulin secretion. It is intriguing that in non-diabetic subjects with a high BMI, the pancreas shows an increased beta-cell mass than in subjects with normal a BMI [33,34,39]. This phenomenon is also observed in mice with hyperinsulinemia [40]. A change in the islet mass or the density of the constituent cell types may affect the paracrine control of islet hormone secretion [41]. Conceptually, the restoration of beta-cell mass in both types of diabetes can normalize the blood glucose level, e.g., islet transplantation could rescue IDDM. Restoration of beta-cell mass could also be achieved by regeneration, i.e., either replicating new cells from preexisting cells or neogenesis of new islets from pancreatic duct epithelial cells [39]. Regeneration of a large number of beta-cells to restore islet function and reverse the diabetic state has been demonstrated in mouse models by the ectopic expression of transcription factors, pancreas and duodenum homeobox protein 1 (PDX1), neurogenin-3, and V-maf musculoaponeurotic fibrosarcoma oncogene homolog A (MafA) [42]. PDX1 is an essential homeodomain transcription factor required for the early embryonic development of the pancreas and the differentiation of pancreatic lineages [43]. No mature beta-cells were found in the fetus of E11.5 with a blockage of PDX1 [44]. In adult mice, impaired PDX1 expression coincided with changes in beta-cell number and insulin secretion and led to the development of diabetes within 14 days. Blood glucose levels returned to normal after the restoration of PDX1 expression (within 28 days). Both PDX1^+^/Insulin^+^ and PDX1^+^/Insulin^−^ cells were stained in duct epithelia, suggesting that PDX1 is a chief factor required for beta-cell neogenesis, differentiation, and maturation [45]. In this study, a large number of PDX1-positive cells were stained in the islets and epithelial cells of the pancreatic duct, suggesting postnatal regeneration of beta-cells in treated db/db mice. We also observed a conversion of alpha-cells to beta-cells. Recent studies supported that alpha-cells may be a potential reservoir for beta-cell regeneration. The overexpression of PDX1 and MafA successfully reprogrammed alpha-cells into beta-cells in vitro and in vivo, providing a significant restoration of islet function and delaying the onset of diabetes [46,47,48]. 

In a previous study, rats with streptozotocin-induced hyperglycemia were orally administered 50, 100, and 150 mg/kg aqueous extract of EL for 10 days, and a reduction in the blood glucose level was only observed with 150 mg/kg of extract (equivalent to 900 mg per day for a 60 kg adult) [49]. However, in this study, antidiabetic effects were not observed in the db/db mice when the aqueous fraction of the suspension was administered instead of the suspension (data not shown). Generally, extracts are considered more toxic than the crude powdered EL root. The safe dose varied among extracts prepared using different solvents. For example, the subacute oral intake of aqueous extract of EL was hepatotoxic when administered to rats at 1200 and 2400 mg/kg [50]. Choudhary and colleagues investigated the subchronic toxicity of the standardized aqueous EL extract (Physta^®^) in Wistar rats, and determined that the no observed adverse effect level (NOAEL) was 1000 mg/kg (the acceptable daily intake [ADI] is 600 mg/day for adults) [51]. Based on these toxicological data, the aqueous extract of EL is generally recommended to be administered at a dose of 200–400 mg daily [11,50]. Therefore, owing to the need to adhere to acceptable safety margins, the antihyperglycemic effect of aqueous extract of EL is unachievable in a clinic. In our study, the antihyperglycemic efficacy was achieved at the dose of 50 mg/kg of crude powdered EL root (equivalent to 300 mg/day for a 60 kg adult). We have previously reported the safety of crude powdered EL root; the NOAEL was found to be a daily dose of 2 g/kg (obtained from a 13-week subchronic toxicity study in rats) and the calculated ADI was up to 1.2 g/adult [24]. Thus, the crude powdered EL root may have value as a remedy. 

## 5. Conclusions

Based on current knowledge, an effective management strategy against the progression of NIDDM should include the simultaneous control of hyperglycemia and dyslipidemia. This study elucidates the antidiabetic activity of EL. Furthermore, EL was found to have beneficial effects on hypercholesterolemia. We are also the first to report that treatment with EL caused an expansion of beta-cell in the islets through PDX1 expression, resulting in the improvement of islet performance. Taken together, EL is a potent traditional medicine that has a satisfactory efficacy in diabetes.

## Figures and Tables

**Table 1:** The mean body weight of db/db mice involving control and powdered *E. longifolia* root treatments.

Powdered *EL* Root (mg/kg)	Mean Body Weight (g)				
	Week 0	Week 1	Week 2	Week 4	Week 8
0	50.65 ± 1.82	52.06 ± 1.57	52.80 ± 1.40	54.4 ± 1.43	55.04 ± 1.61
25	54.89 ± 1.27	55.07 ± 1.24	55.13 ± 1.23	54.49 ± 1.38	56.16 ± 1.21
50	52.91 ± 2.53	53.75 ± 2.17	53.71 ± 1.76	54.15 ± 1.80	53.16 ± 1.87
100	51.54 ± 1.86	51.84 ± 1.80	51.15 ± 2.05	50.58 ± 2.47	50.17 ± 3.05

Mice received sterile water (control group) or powdered *E. longifolia* (EL) root (25, 50, and 100 mg/kg) suspension by gavage once daily. The experimental period was eight weeks. Body weight was recorded every week. Data are expressed as the mean ± S.E.M. (*n* = 10). ANOVA was performed.

**Table 2:** Antihyperglycemic activity of powdered *E. longifolia* root in db/db mice.

Powdered *EL* Root (mg/kg)	Blood Glucose Level (mg/dL)				
	Week 0	Week 1	Week 2	Week 4	Week 8
0	346.2 ± 11.9	367.0 ± 33.2	344.67 ± 26.4	398.1 ± 17.8	373.6 ± 32.0
25		306.0 ± 33.2	305.2 ± 26.3	285.6 ± 35.5	282.3 ± 35.7
50		300.3 ± 38.7	226.6 ± 36.0 *	205.0 ± 26.3 *	193.6 ± 29.7 *
100		228.8 ± 27.7 *	150.8 ± 18.5 *	143.0 ± 16.4 *	134.6 ± 18.7 *

Mice received sterile water (control group) or powdered EL root (25, 50, and 100 mg/kg) suspension by gavage once daily. The experimental period was eight weeks. Blood glucose levels were measured at weeks 0, 1, 2, 4, and 8, as described in Materials and Methods. Data are expressed as the mean ± S.E.M. (*n* = 10). ANOVA was performed. * *p* < 0.05.

**Table 3:** Effects of powdered *E. longifolia* root in an Oral Glucose Tolerance Test (OGTT) in db/db mice.

Powdered EL Root (mg/kg)	Blood Glucose Level (mg/dL)					
	0 min	30 min	60 min	90 min	120 min	180 min
0	373.9 ± 31.0	750.4 ± 43.6	680.5 ± 33.8	555.7 ± 32.9	503.6 ± 33.6	398.2 ± 26.5
25	337.9 ± 46.8	582.5 ± 37.8	584.2 ± 49.2	542.8 ± 46.1	484.5 ± 36.9	395.4 ± 42.9
50	269.5 ± 37.2	523.0 ± 65.7	496.2 ± 71.6	446.8 ± 78.8	427.8 ± 86.6	362.3 ± 57.6
100	174.9 ± 26.5	396.4 ± 23.7	322.3 ± 30.2	279.8 ± 24.7	252.9 ± 25.5	181.4 ± 20.4

OGTT was performed at week 7. Before the experiment, the mice were fasted for 16–20 h and treated with glucose (1 g/kg) by gavage. Blood glucose levels were estimated at indicated time points as described in Materials and Methods. Data are expressed as the mean ± S.E.M. (*n* = 10). ANOVA was performed.

## Abbreviations

EL	Eurycoma longifolia
DM	diabetes mellitus
IDDM	insulin-dependent diabetes mellitus
NIDDM	non-insulin-dependent diabetes mellitus
PDX1	pancreatic and duodenal homeobox 1
HbA1c	hemoglobin A1c
OGTT	Oral glucose tolerance test
NOAEL	no observed adverse effect level
ADI	acceptable daily intake
HOMA-IR	homeostasis model assessment-insulin resistance
HOMA-β	homeostasis model assessment-beta-cell function

## Appendix A

**Table A1:** The anti-hyperglycemia activity of powdered *E. longifolia* root in diabetic db/db mice and normoglycemic C57BL/6J mice (pilot study).

Powdered *EL* Root (mg/kg)	Blood Glucose Level (mg/dL)					
	Diabetic db/db Mice			Normoglycemic C57BL/6J Mice		
	Week 0	Week 1	Week 2	Week 0	Week 1	Week 2
0	361.0 ± 49.0	366.0 ± 46.5	336.3 ± 19.9	128.6 ± 7.1	153.4 ± 6.7	118.2 ± 7.2
200	369.3 ± 20.0	184.8 ± 20.4 *	150.0 ± 22.8 *	156.6 ± 2.2	137.4 ± 5.4	115.8 ± 5.7

Mice were received sterile water (control group), 200 mg/kg and 400 mg/kg suspensions of powdered EL root by gavage once per day. The blood glucose levels were measured as described in Materials and Methods. Animals of the 400 mg/kg treated group were found dead in week 1 (2/4) and week 2 (4/4). After 200 mg/kg treatment for 8 weeks, the mortality was 50% (4/8). The dying animals were weak, and their blood glucose levels were < 100 mg/dL. Data are expressed as the mean ± S.E.M. (*n* = 10). Data were expressed as the mean ± S.E.M. ANOVA was performed. * *p* < 0.05.
